# Supplementary material for: Automated slice‐specific z‐shimming for functional magnetic resonance imaging of the human spinal cord
Source: Hum Brain Mapp. 2022 Aug 8;43(18):5389–407. doi: 10.1002/hbm.26018 (PMC9704784; doi:10.1002/hbm.26018)
Supplement: Supplementary file 1 — Appendix S1 Supplementary Information [file HBM-43-5389-s001.pdf]

Automated slice-specific z-shimming for fMRI of the human spinal cord

Merve Kaptan [a*], S. Johanna Vannesjo [b], Toralf Mildner [a], Ulrike Horn [a], Ronald Hartley-Davies [c], Valeria Oliva [d], Jonathan C.W. Brooks [e], Nikolaus Weiskopf [a,f], Jürgen Finsterbusch [g], Falk Eippert [a*]

[a] Max Planck Institute for Human Cognitive and Brain Sciences, Leipzig, Germany

[b] Department of Physics, Norwegian University of Science and Technology, Trondheim, Norway

[c] Department of Medical Physics, University Hospitals Bristol and Weston, Bristol, United Kingdom

[d] School of Physiology, Pharmacology and Neuroscience, University of Bristol, Bristol, United Kingdom

[e] School of Psychology, University of East Anglia Wellcome Wolfson Brain Imaging Centre (UWWBIC), Norwich, United Kingdom

[f] Felix Bloch Institute for Solid State Physics, Faculty of Physics and Earth Sciences, Leipzig University, Leipzig, Germany

[g] Department of Systems Neuroscience, University Medical Center Hamburg-Eppendorf, Hamburg, Germany

[*] Corresponding authors

Address for correspondence: Merve Kaptan & Falk Eippert; Max Planck Research Group Pain Perception, Max Planck Institute for Human Cognitive and Brain Sciences, Stephanstraße 1a, 04103 Leipzig, Germany, Phone: +49 341 9940 2224; mkaptan@cbs.mpg.de; eippert@cbs.mpg.de

Supplementary Material

Please note that for sake of readability, the numbering of the headers in the Supplementary Material was kept consistent with the Results section in the main text.

3.1 Replication and extension of previous findings

3.1.1 Direct replication

To additionally investigate how robust the findings in the main manuscript are, we supplement the single-volume analyses (that might be affected by various noise sources) by the same analysis approach, but now carried out on an EPI volume that is the average of a time-series of 250 motion corrected EPI volumes (acquired both for no z-shim and manual z-shim). We observed a significant increase of mean signal intensity ($t_{(47)}$ = 19.03, p < .001, difference of 12%) and a significant reduction of signal intensity variation across slices ($t_{(47)}$ = 27.22, p < .001, difference of 51%) for manual z-shim compared to no z-shim.

We also conducted the same analysis in native space (both for single-volume data and the average of a time-series of 250 motion corrected EPI volumes) instead of template space and observed very similar results demonstrating the benefit of z-shimming: for the single-volume data, we observed a significant increase of mean signal intensity ($t_{(47)}$ = 21.07, p < .001, difference of 19%) and a significant reduction of signal intensity variation across slices ($t_{(47)}$ = 25.55, p < .001, difference of 55%). For the average of a time-series of 250 motion corrected EPI volumes, we also observed a significant increase of mean signal intensity ($t_{(47)}$ = 20.14, p < .001, difference of 16%) and a significant reduction of signal intensity variation across slices ($t_{(47)}$ = 26.15, p < .001, difference of 54%). All of these results mirror those reported in the main manuscript.

3.1.2 Slice-by-slice characterization of z-shim effects

Here, we complement the qualitative results reported in the main text by a more formal approach: we first carried out an analysis where we categorized each slice of the single-volume EPIs according to the step-difference between the manually chosen z-shim value and that of no z-shim (value 11) and compared the signal intensity in these categories between no z-shim and manual z-shim using a 2x5 repeated-measures ANOVA (factor 1: *condition* with levels no z-shim and manual z-shim; factor 2: *step-difference* of 0, 1, 2, 3, >3). We observed a significant main effect of condition ($F_{(1,88)}$ = 222.74, p < .001), a significant main effect of step-difference ($F_{(4,352)}$ = 355.8, p < .001) and a significant interaction ($F_{(4,352)}$ = 204.66, p < .001). Post-hoc Bonferroni-corrected t-tests then revealed that the signal intensity improvement by z-shimming was not significant in those slices that had no step-difference and thus served as a negative control ($t_{(47)}$ = 1.16, p = 1), but that it increased with increasing step-difference (step-difference of 1: $t_{(47)}$ = 11.48 p < .001;

step-difference of 2: $t_{(47)} = 19.84$, $p < .001$; step-difference of 3: $t_{(47)} = 18.02$, $p < .001$; step-difference of >3: $t_{(47)} = 35.13$, $p < .001$).

In order to assess the robustness of these effects, we also repeated the same analysis on the average of 250 motion corrected EPI volumes. The ANOVA showed a significant main effect of condition ($F_{(1,88)} = 145.99$, $p < .001$), a significant main effect of step-difference ($F_{(4,352)} = 311.18$, $p < .001$) and a significant interaction ($F_{(4,352)} = 184.72$, $p < .001$). Post-hoc Bonferroni-corrected t-tests revealed that the signal intensity in the no z-shimming condition unexpectedly was minimally (but consistently) higher in those slices that had no step-difference control ($t_{(47)} = -4.95$, $p < .001$, difference of 1%), but more importantly that the beneficial effect of z-shimming increased with increasing step-difference (step-difference of 1: $t_{(47)} = 5.35$ $p < .001$, difference of 3%; step-difference of 2: $t_{(47)} = 16.91$, $p < .001$, difference of 16%; step-difference of 3: $t_{(47)} = 13.45$, $p < .001$, difference of 32%; step-difference of >3: $t_{(47)} = 32.79$, $p < .001$, difference of 115%).

3.1.3 z-shim effects across different TEs

When we repeated the analysis from the main text on the average of 25 motion-corrected volumes we observed very similar results. The effects of z-shimming were highly significant both at the TE of 30ms (mean signal intensity: $t_{(47)} = 21.40$, $p < .001$, difference of 10%; signal intensity variation across slices: $t_{(47)} = 22.60$, $p < .001$, difference of 48%) and at the TE of 50ms (mean signal intensity: $t_{(47)} = 16.70$, $p < .001$, difference of 12%; signal intensity variation across slices: $t_{(47)} = 20.80$, $p < .001$, difference of 44%).

3.1.4 z-shim effects in gray matter regions

In order to formally compare the mean of signal intensity in different gray matter regions, we used a 2x2 repeated-measures ANOVA (factor 1: *condition* with levels no z-shim and manual z-shim; factor 2: *anatomical location* with levels dorsal horn and ventral horn). We observed a significant main effect of condition ($F_{(1,94)} = 621.33$, $p < .001$), a significant main effect of anatomical location ($F_{(1,94)} = 39.70$, $p < .001$) and a significant interaction ($F_{(1,94)} = 21.31$, $p< .001$); note that post-hoc t-tests are reported in the main text.

When we investigated the variation of signal intensity using the same ANOVA approach, we observed a significant main effect of condition ($F_{(1,94)} = 1024.40$, $p < .001$), a significant main effect of anatomical location ($F_{(1,94)} = 30.32$, $p < .001$) and a significant interaction ($F_{(1,94)} = 9.60$, $p = .003$). Following this up with post-hoc Bonferroni-corrected t-tests revealed there was a significant beneficial effect of z-shimming in the dorsal horn ($t_{(47)} = 21.43$, $p < .001$, difference of 48%), as well as in the ventral horn ($t_{(47)} = 25.18$, $p < .001$, difference of 47%), but that the beneficial effect of z-shimming was more evident in the dorsal horn than in the ventral horn ($t_{(47)} = 4.06$, $p < .001$).

As a negative control analysis, we carried out the same ANOVA approach, but now using the mean of signal intensity from the left vs right parts of the cord, where no differential effects should occur. As expected, we observed a significant main effect of condition ($F_{(1,94)} = 690.05$, $p < .001$), no significant main effect of location ($F_{(1,94)} = 0.01$, $p = 0.90$) and no significant interaction ($F_{(1,94)} = 0.09$, $p = 0.76$).

We repeated the above analyses (which are based on single-volume EPIs) with an average of the 250 motion-corrected volumes. A 2x2 repeated-measures ANOVA (factor 1: *condition* with levels no z-shim and manual z-shim; factor 2: *anatomical location* with levels dorsal horn and ventral horn) showed a significant a significant main effect of condition ($F_{(1,94)} = 629.52$, $p < .001$), a significant main effect of anatomical location ($F_{(1,94)} = 36.55$, $p < .001$) and a significant interaction ($F_{(1,94)} = 4.90$, $p = .03$). Post-hoc Bonferroni-corrected t-tests revealed there was a significant beneficial effect of z-shimming in terms of the signal intensity in the dorsal horn ($t_{(47)} = 18.85$, $p < .001$, difference of 15%), as well as in the ventral horn ($t_{(47)} = 16.59$, $p < .001$, difference of 11%), but that the beneficial effect of z-shimming was more evident in the dorsal horn than in the ventral horn ($t_{(47)} = 4.87$, $p < .001$). With respect to variation of signal intensity, the ANOVA resulted in a significant main effect of condition ($F_{(1,94)} = 1300.20$, $p < .001$), a significant main effect of anatomical location ($F_{(1,94)} = 27.78$, $p < .001$) and a significant interaction ($F_{(1,94)} = 4.08$, $p = .046$). Post-hoc Bonferroni-corrected t-tests revealed there was a significant beneficial effect of z-shimming in terms of reduction in the signal intensity variation over slices in the dorsal horn ($t_{(47)} = 24.95$, $p < .001$, difference of 46%), as well as in the ventral horn ($t_{(47)} = 26.33$, $p < .001$, difference of 48%), but that the beneficial effect of z-shimming was more evident in the dorsal horn than in the ventral horn ($t_{(47)} = 2.69$, $p = 0.01$).

3.1.5 z-shim effects on time-series data

When we investigated the effects of z-shimming on tSNR using motion-censored time-series data, we observed a significant increase in mean tSNR ($t_{(47)} = 10.73$, $p < .001$, difference of 9%), as well as a significant reduction of tSNR variation across slices ($t_{(47)} = 10.94$, $p < .001$, difference of 25%). In the most-affected slices, z-shimming increased the tSNR by 26% on average, ranging from 6% to 116% across participants (this analysis revealed that there were 3 outliers where tSNR decreased by 1% (for two of the outliers) and 29% (for one of the outliers) for manual z-shimming compared to no z-shimming), again similar to what is reported in the main manuscript.

3.2 Automation of z-shimming

3.2.1 EPI-based automation

When analyzing single-volume EPI gray matter signal intensity (in order to relate these effects to those from the direct replication performed earlier), we observed a significant increase of mean signal intensity ($t_{(23)} = 12.51$, $p < .001$, difference of 13%) and a significant decrease in signal

intensity variation across slices ($t_{(23)} = 16.89$, $p < .001$, difference of 51%) for manual z-shimming against no z-shimming. Most importantly, we found a similarly beneficial effect when using our automated approach, i.e. a significant increase in mean signal intensity ($t_{(23)} = 12.18$, $p < .001$, difference of 14%) and a significant decrease in signal intensity variation across slices ($t_{(23)} = 16.97$, $p < .001$, difference of 48%). When directly comparing the two approaches to determine z-shim values, we observed no significant difference, neither for mean signal intensity ($t_{(23)} = 0.31$, $p = 1$), nor for signal variation across slices ($t_{(23)} = 2.49$, $p = 0.06$), though in both cases the performance of the automated approach was slightly superior. Overall, these results strongly mirror those based on tSNR reported in the main manuscript.

3.2.2 Field map based (FM-based) automation

When analyzing single-volume EPI gray matter signal intensity, we observed a significant increase of mean signal intensity ($t_{(23)} = 15.39$, $p < .001$, difference of 15%) and a significant decrease in signal intensity variation across slices ($t_{(23)} = 20.81$, $p < .001$, difference of 52%) for manual z-shimming against no z-shimming. Most importantly, we found a similarly beneficial effect when using our automated approach, i.e. a significant increase in mean signal ($t_{(23)} = 13.59$, $p < .001$, difference of 12%) and a significant decrease in signal variation across slices ($t_{(23)} = 17.42$, $p < .001$, difference of 49%). When directly comparing the two approaches to determine z-shim values, we observed a significant difference for the mean signal intensity ($t_{(23)} = 3.82$, $p = 0.003$), but not for variation across slices ($t_{(23)} = 1.52$, $p = 0.43$), again showing the slightly inferior performance of this automated approach compared to manual z-shimming, congruent with the tSNR-based results in the main manuscript.

In the following, we detail the post-hoc investigations we undertook in order to determine possible reasons for the unexpected sub-optimal performance of the FM-based approach. Briefly, we first used the vendor-based field map and assessed the contributions of i) the choice of mask for identifying the spinal cord in the field map phase data, ii) various choices of parameters employed in the fitting process of the gradient field, iii) field-gradients in the AP-direction, and iv) inhomogeneity-induced mis-localizations between EPIs and field map. Second, we substituted the vendor-based field map by the more robust in-house field map and compared their performance. Third, we assessed the general reliability of estimating z-shim values from field map data by repeating the fitting process on a second in-house field map that was acquired at the end of the experiment. Finally, we calculated the optimum z-shim values using a histogram-based evaluation instead of a linear fit to reduce the influence of extreme values. In order to determine whether the performance of FM-based z-shim selection would improve with the different post-hoc approaches we undertook, we i) calculated the chosen z-shim values, ii) based on those we then artificially 'reconstructed' the EPI z-shim reference scan for each approach (see Methods section 2.7.3 for details), and iii) compared the gray matter signal characteristics (*mean* and *coefficient of variation*) between the new implementation and the original implementation. Please note that since we have

a directional hypothesis (new FM-approach better than main manuscript FM-approach), we only test for an improvement compared to our original implementation.

3.2.2.1 Choice of mask for identifying the spinal cord in the field map phase data

In our original implementation, the fitting of the linear gradient field was performed only on voxels within the spinal cord. This voxel selection was determined by a mask that was obtained from a segmentation of the T2-weighted image. While visual inspection of the mask overlaid onto the field map magnitude image did not give cause for concern in any of the 48 participants (i.e. due to possible participant movement between T2 and field map acquisitions), we nevertheless asked whether a change of the mask might improve performance. We therefore re-ran the original fitting procedure, but now based on a mask that was either eroded by 1 voxel or dilated by 1 voxel. When comparing the results based on these new masks to the standard mask, we observed that neither of these changes resulted in a meaningful and significant change in gray matter mean signal intensity (11% increase against no z-shim for all three masks) or signal intensity variation across slices (50% decrease for original and dilated masks, 49% decrease for eroded mask compared to no z-shimming). In line with these descriptive results, when directly comparing the original and new approaches statistically, we observed no significant differences (all $p_{uncorrected} > 0.30$).

3.2.2.2 Choice of parameters employed in the fitting process of the gradient field

In our original implementation, we chose the following parameters based on pilot acquisitions: we smoothed the field map data with an isotropic 1mm kernel, used 9mm slab thickness (i.e. 9 transversal field map slices) for each fit and gave equal weight to all voxels in the fitting procedure. We next investigated whether variations of these parameters might have an influence on the performance: the smoothing kernel width (sigma) was set to 0, 1 or 2mm; the slab thickness was set to 5, 9 or 13mm, either with equal weighting or weighted by a raised cosine kernel of full-width half-maximum equal to the slab thickness and a roll-off factor beta of 0.5 (the purpose of the weighting was to down-weight voxels further away from the corresponding EPI slice). However, none of these choices seemed to make a meaningful difference, although out of these 17 additional variations (3 smoothing options crossed with 3 slab-thickness options and 2 weighting options) one showed a slight improvement for mean signal intensity and one showed a slight improvement for signal intensity variation along slices compared to our initial parameter set of choice that we used throughout the experiment (maximum improvement for mean signal intensity observed with parameter set "slab thickness = 9, beta = 0.5, smoothing sigma = 1": $t_{(47)} = 1.64$, $p_{uncorreced} = 0.054$, $p_{corrected} = 1$, difference of 0.2%; maximum improvement for signal intensity variation over slices observed with parameter set "slab thickness = 9, beta = 0, smoothing sigma = 0": $t_{(47)} = 2.92$, $p_{uncorrected} = 0.003$, $p_{corrected} = 0.05$, difference of 2%).Thus, the slightly worse performance of the FM-based approach reported in the main manuscript – which was based on a significant difference

for mean signal intensity – does not seem to be due to the choice of parameters employed in the fitting process.

3.2.2.3 Field gradients in the AP-direction

Another possible explanation for the slightly inferior performance of the FM-based approach is that field inhomogeneities in the A-P (y) direction may shift the center of k-space in the EPI acquisitions which – depending on their polarity – would result in a shorter or longer effective TE. Because the calculation of the required z-shim gradient moment from the field map assumes that the echo forms up at the nominal TE, any shift of the effective TE would lead to an imperfect compensation of the through-slice dephasing and would cause a signal loss. This is in contrast to the EPI-based approach, which rests on an EPI acquisition where the effective TE is inherently considered by just picking the best z-shim moment tested.

In the presence of a susceptibility-induced field gradient in the phase encoding direction, G_{SP}, refocusing happens at an effective TE given by:

$$TE_{eff} = \frac{TE}{Q},$$

where

$$Q = 1 - \frac{G_{SP}}{G_{PE}}.$$

G_{PE} is the effective phase encoding gradient:

$$G_{PE} = \frac{1}{\gamma} \cdot \frac{dk}{dt},$$

where dt is the echo spacing (Deichmann et al., 2002). Based on the fitted linear field gradient in the AP direction, we calculated Q for each slice, and adjusted the z-shim gradient moment to account for the effective TE. We then investigated how the adjustment of the z-shim moments (please note that we considered both positive and negative polarities of EPI phase-encoding) affected the gray matter signal characteristics compared to our original implementation. We neither observed a meaningful increase in mean signal intensity (negative polarity vs original implementation: $t_{(47)} = -0.59$, $p_{uncorreced} = 0.72$, $p_{corrected} = 1$, <0.1% decrease; positive polarity vs original implementation: $t_{(47)} = 0.08$, $p_{uncorreced} = 0.47$, $p_{corrected} = 0.94$, <0.1% increase) nor a meaningful decrease in signal intensity variation over slices (negative polarity vs original implementation: $t_{(47)} = -0.93$, $p_{uncorreced} = 0.82$, $p_{corrected} = 1$, 0.8% increase; positive polarity vs original implementation: $t_{(47)} = -1.58$, $p_{uncorreced} = 0.06$, $p_{corrected} = 0.12$, 1% decrease). It thus seems that the influence of AP gradients is rather negligible with respect to the slightly inferior performance of the FM-based approach.

3.2.2.4 Inhomogeneity-induced mis-localizations between EPIs and field map

The FM-based z-shim selection relies on spatial congruency between the field map and the EPI acquisitions in the through-slice direction of the axial EPI volume. Local susceptibility-induced field offsets can however affect the spatial congruency in different ways. In the sagittal field map acquisitions, local field offsets will result in a shift along the readout direction, i.e. superior-inferior. The readout bandwidth of the field map acquisition was 630 Hz/pixel at a voxel resolution of 1 mm. Except for the region most inferior, the local field offsets were below 100 Hz, which results in voxel shifts of less than 0.16 mm. In the EPI-acquisitions, the field offset affects the effective slice localization. The EPI excitation bandwidth was ~2 kHz at a 5 mm slice thickness. Field offsets <100 Hz would thus correspond to slice shifts <0.25 mm. In the worst-case scenario, where both effects are superimposed, an effective relative spatial shift of <0.4 mm is obtained which was deemed small enough to have a negligible impact on the z-shim selection.

However, to additionally empirically investigate whether an inhomogeneity-induced mis-localization between the EPI and the field map might be a driving factor for the slightly inferior performance of the FM-based approach, we selected the participants for which the FM-based automated selection of z-shim values led to a step-size difference of at least three steps in at least one slice compared to the manual z-shim values (N = 10). In other words, we tried to identify a sub-group with the most extreme differences, since higher step size differences compared to manual z-shim implies that the field map selection of z-shim values was unsuccessful or 'off'. In those participants, we plotted the local field offset and the absolute difference between automated and manually selected z-shim values (Supplementary Figure 4) and then visually investigated whether there would be any detectable relationship between a high step size difference and high field offset. However, these plots do not indicate that higher step size differences generally coincide with high local field offsets.

3.2.2.5 Use of different field map

We also investigated whether the quality of our default field map protocol might have led to the slightly inferior performance of the FM-based approach. In order to assess this, we calculated z-shim values not only based on the originally chosen field map (vendor-provided with 2 echoes), but also based on a separate in-house field map (with 12 echoes) which was acquired directly after the vendor-based one. When we quantified the signal characteristics, we observed that both methods led to a similar increase in mean signal intensity (11% for vendor-based and 12% for in-house field map) and decrease in signal intensity variation across slices (50% for vendor-based field map and 52% for in-house field map). In line with these descriptive results, when directly comparing the performance of the vendor-based and in-house approaches statistically (e.g. auto-vendor compared to auto-in-house; note that the baseline of no z-shimming is identical between the two), we observed a slight benefit of the in-house field map (for mean signal intensity: $t_{(47)}$ = 2.34, p = 0.01, difference of 0.5%, for signal intensity variation over slices: $t_{(47)}$ = 1.84, p = 0.04,

difference of 4%). In order to test whether this improvement would lead to a change in the pattern reported in the main text (i.e. the FM-based approach performing worse when comparing mean tSNR for the automated compared to the manual approach), we followed up on this by comparing the performance of both approaches against manual approach and still observed a slightly inferior performance for FM-based approaches for the mean signal intensity (auto-vendor compared to manual: $t_{(47)} = 7.14$, $p < .001$, difference of 2.0%; auto-in-house compared to manual: $t_{(47)} = 8.20$, $p < .001$, difference of 1.5%) but not for the coefficient of variation (auto-vendor compared to manual: $t_{(47)} = 0.46$, $p = 0.65$; auto-in-house compared to manual: $t_{(47)} = 1.40$, $p = .17$), which is consistent with the results reported in the main text.

3.2.2.6 Assessing the reliability of z-shim selection based on FM-based automation

To probe how reliably z-shims can in general be determined via field maps, we also acquired a second in-house field map near the end of our experiment (please note that due to technical problems the second field map was not acquired for three participants) and investigated whether this would result in similar automatically chosen z-shim values: across participants, we observed a mean Spearman rank-correlation of rs = 0.88, range: 0.50-0.98), suggesting that the robustness of the FM-based determination is unlikely to be a driving factor in the slightly inferior performance.

3.2.2.7 Evaluating a histogram-based method of determining z-shims

In a further approach, we used a histogram-based method for automatically determining the slice-specific z-shim values from the field map data. This was based on the idea that for a broad distribution of field inhomogeneities, the chosen compensation gradient may only be able to recover significant signal for those voxels with a field inhomogeneity similar to that perfectly compensated. For a skewed distribution, the mean value may be shifted towards inhomogeneities that are less frequent which may reduce the overall signal accordingly. Thus, the chosen approach was based on the histogram of inhomogeneities and considered particularly the most frequent values. We first calculated the B_0 z-gradient for all x- and y-values of each 1mm sub-slice of the vendor-based B_0 map (swapped to the orientation of the EPI space) using the IDL procedure “gradient.pro” which, after proper scaling, resulted in a $gradB_{0,z}$ map of the same resolution as the B_0 map in mT/m. A histogram of $gradB_{0,z}$ was then calculated for each EPI slice in a region-of-interest containing the spinal cord (as with all FM-based procedures, this was obtained from a segmentation of the T2-weighted image) of all of its five 1-mm sub-slices using a bin size of 0.01 mT/m. The resulting histogram was then smoothed with a kernel width of 1/20 of the total number of bins. Next, the main peak in the histogram was determined by comparing the surrounding of the three most frequent bins using the average of the respective center +/- 2 points. The final processing step for calculating a z-shim value for each EPI-slice was an weighted summation of the $gradB_{0,z}$ bins within the range of the center +/- 10 points (corresponding to +/- 0.1 mT/m)

around the resulting main peak with the constraint that the actual summation range was limited to points possessing more than 25% of the center's intensity.

To demonstrate the improvement afforded by this method, we first show data from a single participant in which the original FM-based automation worked poorly in several slices. The left panel of Figure 6A shows that in an exemplary problematic slice, the z-gradient of the B_0 map is not homogeneous across the spinal cord, leading to an asymmetric distribution of z-gradients and a sub-optimal choice of the z-shim value for this slice if the original approach is used (green line), differing also from the z-shim value determined by manual or EPI-based selection. Using the histogram-based method described above, the most probable value of $gradB_{0,z}$ (gray line) is obtained by the intensity-weighted summation of the histogram around the main peak. As a consequence, the z-shim value obtained by this method better fits to that of the manual or EPI-based selection. In slices with more homogenous z-gradients across the spinal cord cross-section, both the original method and the histogram method provide virtually identical results (Figure 6A right panel).

We also assessed the improvement in signal quality offered by this method at the group-level, where we used the above-mentioned 'reconstruction' of the z-shim reference scan of each participant, using the slice-specific z-shim values suggested by the histogram-based method. Figure 6B shows that while this method did not completely eliminate the inferior performance of the FM-based approach, it led to a substantial improvement in signal quality across the group. When directly comparing the performance of the original FM-based approach to the histogram-based approach statistically, we observed a significant benefit of the histogram-based approach (mean signal intensity: $t_{(47)} = 5.05$, $p < .001$, difference of 1.3%; signal intensity variation over slices: $t_{(47)} = 0.17$, p = n.s.). We then followed up on this by comparing the performance of the histogram-based approach against the manual approach and still observed a slightly inferior performance for FM-based approach for the mean signal intensity (histogram-based compared to manual: $t_{(47)} = 4.05$, $p < .001$, a difference of 0.75%) but not for the coefficient of variation, in line with previous results. This minor penalty of the FM-based approach may be related to the fact that the relative signal intensities of the individual voxels as they contribute to the EPI image were not considered – this is in contrast to the approach based on the EPI reference scan. Together, this demonstrates that the evaluation of $gradB_{0,z}$ by considering the corresponding histograms is capable of reducing the error in FM-based z-shim selection, even if it does not reach the performance of the manual approach.

3.2.3 Comparing all three approaches

In order to compare how close the automated and manual (current 'gold standard') shim selection processes were, we calculated rank-based correlations and Euclidian distances between the chosen z-shim values in each of the two groups of 24 participants. This was done on a participant-by-participant basis for both metrics, which were based on the same input: slice-wise (i.e. 24) z-shim

values from 1 to 21 (with 11 designating the neutral state of no z-shim) in the manual condition and in an automated condition. In both cases, we used two-sample t-tests to compare the two sub-groups (i.e. EPI-based automation and FM-based automation).

First, we calculated rank-based correlations between the values chosen for the manual and the automated approach. We observed very high correlations of z-shim values in the EPI-based group (average correlation: $r_s = 0.95$, $t_{(23)} = 33.28$, $p < .001$; range of correlations across participants: 0.85 - 0.99), as well as in the FM-based group (average correlation: $r_s = 0.91$, $t_{(23)} = 26.04$, $p < .001$; range of correlations across participants: 0.62 - 0.97). When directly comparing the two groups, we observed that the correlations were significantly higher in the EPI-based group ($t_{(46)} = 2.67$, $p = .01$).

Second (and overcoming the inherent limitations of a correlation-based approach, i.e. the fact that a perfect correlation might be obtained if the pattern of z-shim values were the same across slices, even if there was a constant shift in z-shim values), we employed the Euclidean distance – the square root of the sum of squared differences between the corresponding elements of the two vectors of z-shim values across slices – between the values chosen for the manual and the automated approach. We observed that while the average Euclidean distance for the EPI-based group was 3.40 (range across participants: 2.24–4.80), it was 5.21 for the FM-based group (range across participants: 3.61– 8.94), leading to a significant difference ($t_{(46)} = 7.19$, $p < .001$).

3.3 Validation of EPI-based automation approach

In the independently acquired data set, we observed that for gray matter signal intensity, manual z-shimming resulted in a significant increase in mean signal intensity ($t_{(112)} = 22.04$, $p < .001$, difference of 25%) and a significant decrease in signal intensity variation across slices ($t_{(112)} = 8.29$, $p < .001$, difference of 40%). When we investigated the performance of our automated EPI-based selection approach, we observed a significant increase in mean signal intensity ($t_{(112)} = 28.44$, $p < .001$, difference of 32%) and a significant decrease in signal intensity variation across slices ($t_{(112)} = 10.32$, $p < .001$, difference of 47%). When we directly compared the automated and manual approaches, we observed that the automated method outperformed the manual method both for mean signal intensity ($t_{(112)} = 12.14$, $p < .001$) and for signal intensity variation across slices ($t_{(112)} = 5.63$, $p < .001$).

Supplementary Figures

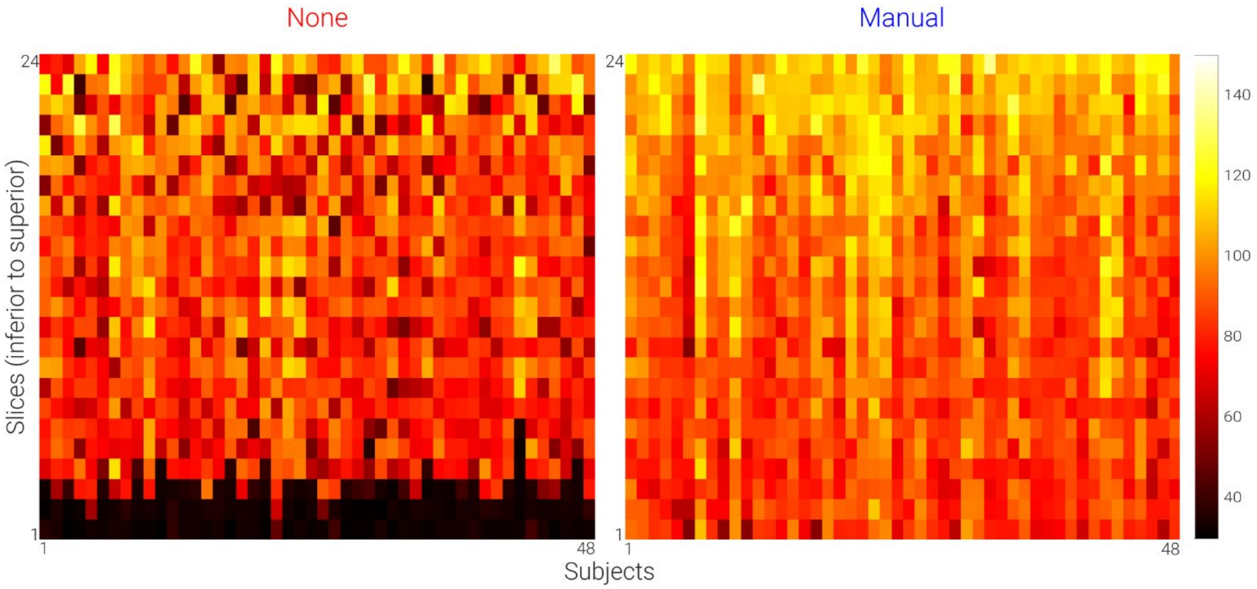

Supplementary Figure 1. **Slice-wise individual signal intensity data.** Based on single volume EPIs acquired without z-shim and with manual z-shim, we calculated the mean signal intensity of each slice in native space. The heat-maps show signal intensity in axial slices (y-axis; 24 slices) for each participant (x-axis; 48 participants).

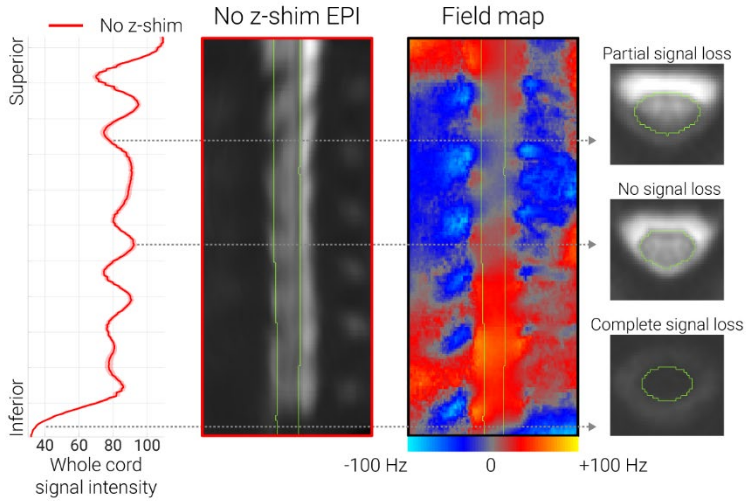

Supplementary Figure 2. **Relationship between field-variations and EPI signal loss**. The line graph on the very left shows the group-averaged (N = 48) template-space spinal cord signal intensity along the rostro-caudal axis of the cord in acquisitions without z-shimming. The solid line depicts the group-mean value and the shaded area depicts the standard error of the mean. The mid-sagittal section on the left shows the group-average template-space single-volume EPI data acquired without z-shimming. The mid-sagittal section on the right shows the group-average template-space field map in order to depict the consistent field variations along the rostro-caudal axis of the cord. On the very right, there are three exemplary axial sections from the "no z-shim' group-average template-space EPIs in order to demonstrate the influence of field variations on the EPI image quality in terms of signal loss.

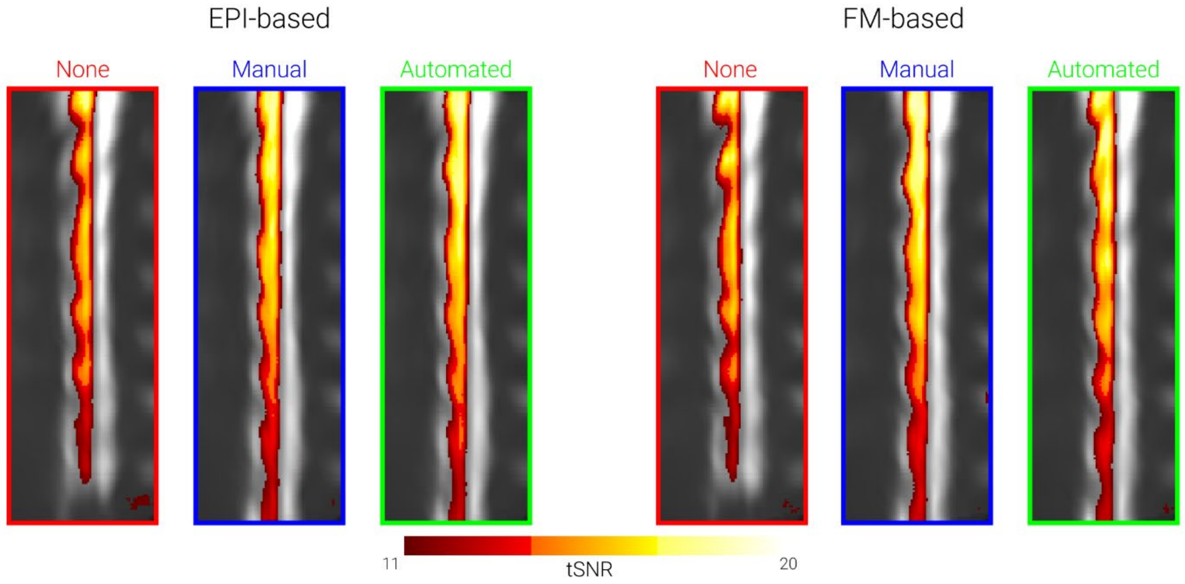

Supplementary Figure 3. **tSNR for different sequence variants.** The mid-sagittal EPI sections in the background consist of the group-average mean of motion-corrected time-series data in template space for each sub-group of participants (EPI-based and FM-based, each of those with N=24) and condition (no z-shim, manual z-shim, automated z-shim). Condition-wise group-average tSNRmaps (based on the motion-corrected EPI data) are overlaid onto these mid-sagittal images (depicted tSNR range: 11-20).

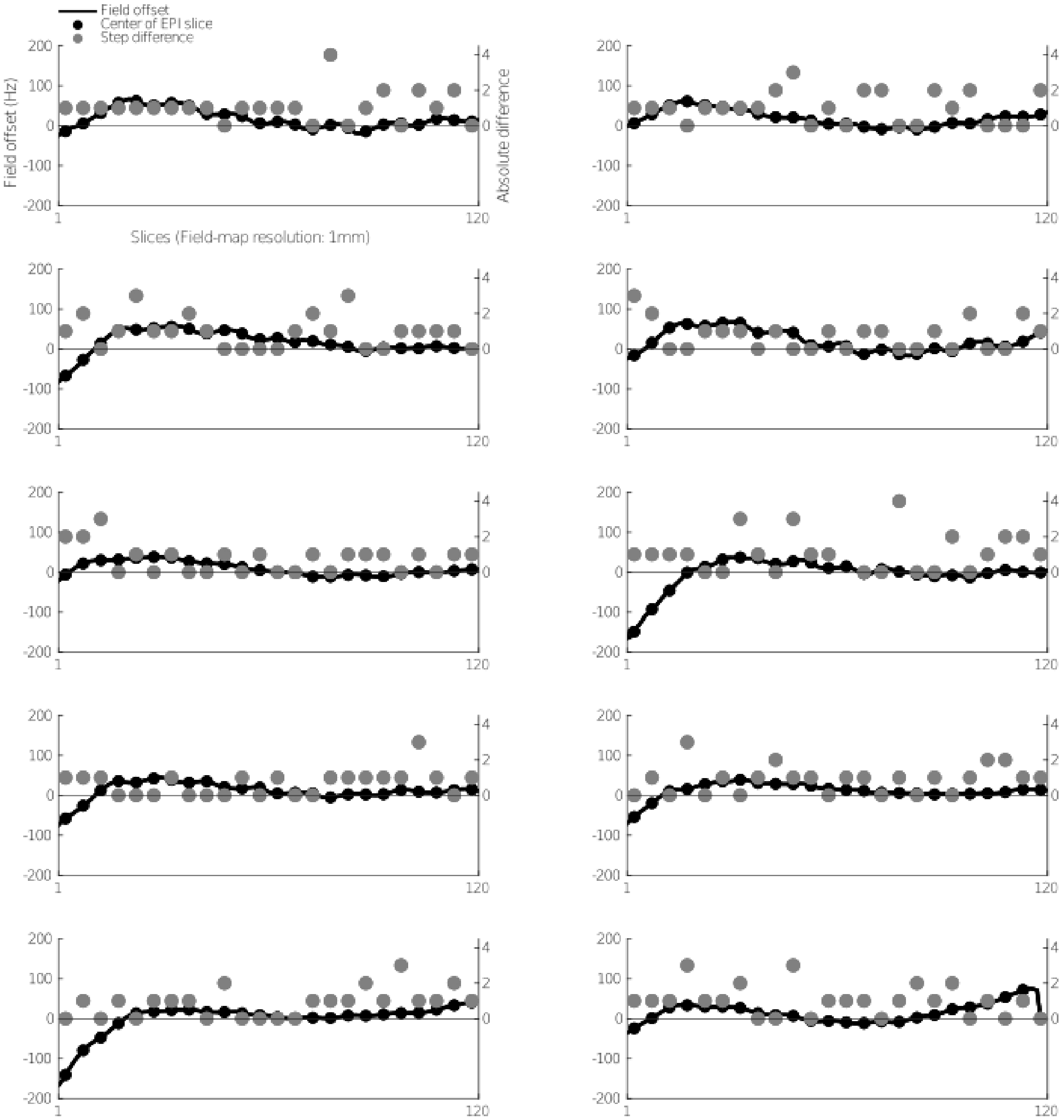

Supplementary Figure 4. **Relationship between field-offset and differential z-shim indices.** Each subplot shows the field offset in Hz (black line; plotted on left y-axis) and the absolute difference in z-shim indices between the FM-based and the manual z-shim selection (gray circles; plotted on right y-axis). Depicted are those participants who had a difference of at least 3 steps between the FM-based and the manual z-shim selection (N = 10). Five FM slices (120 slices in total, 1mm slice thickness) correspond to a single EPI slice (24 slices in total, 5 mm slice thickness) with the black filled dots representing the corresponding center of each EPI slice in the FM resolution.

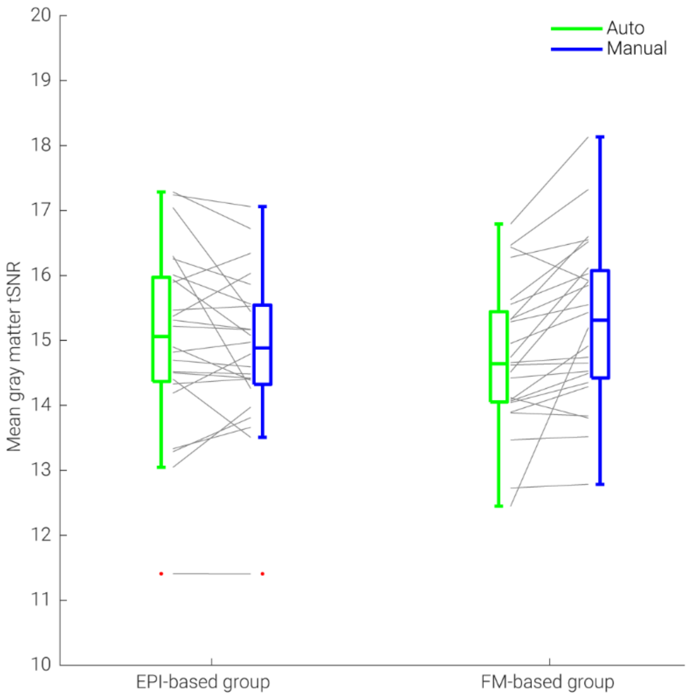

Supplementary Figure 5. **Mean gray matter tSNR of automated and manual approaches.** We calculated the mean gray matter tSNR based on motion-corrected time series data acquired with different sequences (N = 24 for each group). The median is denoted by the central mark and the bottom and top edges of the boxes represent the 25th and 75th percentiles, respectively. The whiskers encompass approximately 99% of the data and outliers are represented by red dots. The gray lines indicate participant-specific tSNR in each condition and its change across conditions.

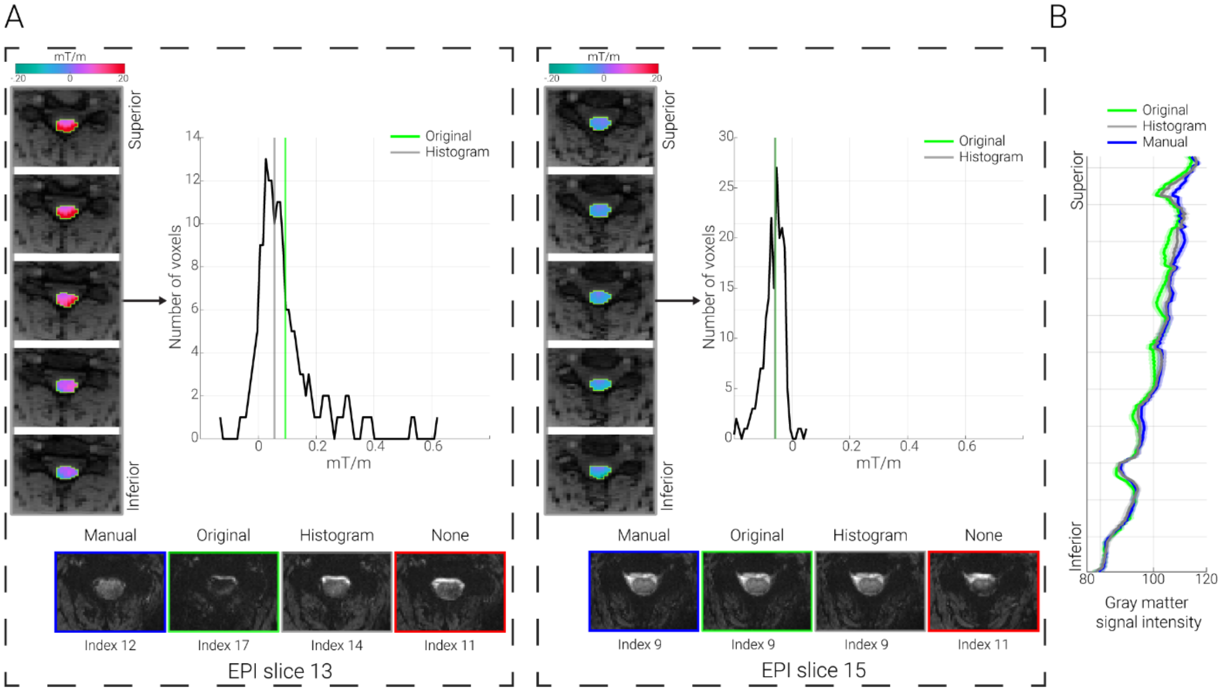

Supplementary Figure 6. **Histogram-based evaluation of field-map. A. Exemplary problematic & unproblematic slices.** In both panels, five axial slices show the gradient map (grad$B_{0,z}$) overlaid on the first magnitude image (for participant ZS030; in native space) corresponding to one EPI slice (problematic slice 13 and unproblematic slice 15, for left and right panels, respectively). The outlines of the cord mask (based on the T2-weighted image) are marked by green lines. The histograms show the grad$B_{0,z}$ for these slices. On the lowermost part, the EPI volumes (corresponding to the selected z-shim indices) from the first z-shim reference image were taken for manual selection, original implementation, histogram-based implementation, and no z-shim condition for the relevant EPI slice. For slices with substantial field variation (problematic slice 13) the histogram-based shim offset selection offers clear improvement over the original automated approach. **B. Group-level signal intensity.** The line graph shows the group-averaged (N = 48) template-space spinal cord signal intensity along the rostro-caudal axis of the gray matter in the reconstructed EPIs (normalized) based on original FM-based implementation (green line), the manual selection (blue line), and based on histogram-based evaluation. The solid lines depict the group-mean value and the shaded areas depict the standard error of the mean.

References

Deichmann, R., Josephs, O., Hutton, C., Corfield, D.R., Turner, R., (2002). Compensation of Susceptibility-Induced BOLD Sensitivity Losses in Echo-Planar fMRI Imaging. *NeuroImage 15*, 120–135. https://doi.org/10.1006/nimg.2001.0985
